# Morphia as a Parturient

**Published:** 1871-04

**Authors:** George H. Vance

**Affiliations:** Swedona, Illinois


### Correspondence.

### MORPHIA AS A PARTURIENT.

SWEDONA, ILLINOIS, *March 15, 1871.*

PROF. N. S. DAVIS,—DEAR SIR,—In your last issue of THE EXAMINER, I notice a communication from L. D. Robinson, M.D., on the "Action of Morphia Sulphatis as a Parturient," in which he quotes largely from an article of Dr. Robson, in the January number of the same journal. For another among many I can say, that so far as my practice has extended, my observations coincide with those of Dr. Robson, and I do invariably believe the action of the sulphate of morphia to be similar to the secale cornutum, whether given in a state of natural or unnatural parturiency.

After the last quotation from Dr. Robson, Dr. Robinson asserts, that in his field of observation, morph. sulph. would prevent *all cases* of threatened abortion, *where correctly administered, that were preventable.* But in *all my* cases where threatened abortion has occurred and I have administered this salt, the parturient throes were immediately increased, and delivery was the result. The same contractile efforts of the womb have been brought about in cases of natural labor, where cessation of action had taken place.

GEO. H. VANCE, M.D.

